# Activation of the TGF-*β* Pathway Enhances the Efficacy of Platinum-Based Chemotherapy in Small Cell Lung Cancer Patients

**DOI:** 10.1155/2022/8766448

**Published:** 2022-12-21

**Authors:** Anqi Lin, Lingxuan Zhu, Aimin Jiang, Weiming Mou, Jian Zhang, Peng Luo

**Affiliations:** ^1^Department of Oncology, Zhujiang Hospital, Southern Medical University, Guangzhou, 510282 Guangdong, China; ^2^The First Clinical Medical School, Southern Medical University, 1023 Shatai South Road, Guangzhou, 510515 Guangdong, China; ^3^Department of Urology, Changhai Hospital, Naval Medical University (Second Military Medical University), Shanghai, China

## Abstract

**Background:**

Platinum-based chemotherapy is the first choice of treatment for patients diagnosed with small lung cell cancer (SCLC). However, many patients exhibit resistance to it. Therefore, it is imperative to further investigate a prognostic biomarker indicating sensitivity to this therapy.

**Methods:**

We collected and performed RNA sequencing on 45 SCLC samples from the Zhujiang Hospital (Local-SCLC). In addition, we used a public cohort from George et al. as a validation cohort (George-SCLC). The transforming growth factor *β* signaling pathway (TGFB) activation status was determined according to the related ssGSEA score. We analyzed immune cell ratios, pathway activation scores, and immune-related genes in SCLC patients to further elucidate the potential mechanisms.

**Results:**

A high activation status of the TGFB pathway was associated with improved prognosis in SCLC patients receiving platinum-based chemotherapy (Local-SCLC: HR = 0.0238, (95% CI, 0.13-0.84), *p* = 0.0238; George-SCLC: HR = 0.0315, (95% CI, 0.28-0.98), *p* = 0.0315). Immune infiltration analysis showed that the TGFB-HIGH group had more M1 macrophages and Th1 cells, whilst fewer M2 macrophages, Th2 cells, and Treg cells were found in the Local-SCLC cohort. Mechanistic analysis showed that the TGBF-HIGH group was upregulated in STING-mediated immunity, apoptosis, and cell cycle arrest, as well as being downregulated in the process of DNA damage repair.

**Conclusions:**

SCLC patients exhibiting a high activation status of the TGFB pathway demonstrate an improved prognosis with platinum-based chemotherapy. The potential underlying mechanism may be related to antitumor immune enhancement and DNA damage repair inhibition.

## 1. Introduction

Lung cancer is the second most prevalent cancer (11.4%) and the leading cause of cancer-related deaths worldwide (18%) [1]. Small cell lung cancer (SCLC) is defined as a rare type of lung cancer, accounting for the presence of approximately 15% of all lung tumors [2, 3]. As an aggressive neuroendocrine tumor, its rapid progression and early metastasis give SCLC an unusually high mortality rate compared to other common solid tumors, as observed in the very low 5-year survival rate (5–10%), and an estimated 200,000 deaths worldwide each year [4–6]. Clinical progress in the treatment of SCLC patients has been slow, with platinum-based chemotherapy being the most common current treatment for a majority of SCLC patients [7]. Unfortunately, although most SCLC patients exhibit a good response to chemotherapy within the early stages of their treatment course, more than 90% of patients develop resistance to chemotherapy and subsequently eventually die from tumor recurrence [8–10]. Therefore, in order to improve the chemotherapy prognosis of SCLC patients, it is vitally important to identify the biomarker that indicates the sensitivity of patients to platinum-based chemotherapy and further explore the possible underlying mechanisms associated therewith.

Resistance to platinum-based chemotherapy in SCLC patients can be caused by many factors. For example, decreased drug absorption, increased drug output, enhanced drug metabolism, and increased DNA damage repair can all lead to resistance to platinum-based chemotherapy, resulting in a poorer prognostic outcome in SCLC patients [11, 12]. Currently, the impact of the tumor immune microenvironment (TIME) on the efficacy of chemotherapy gradually receives increased attention [13, 14]. Studies have shown that the antitumor effects of chemotherapy are mediated in part by the activation of the immune system [15]. Xu et al. found that the activation of the T cell immune response could reverse the chemoresistance of tumors [16]. In addition, immune cell infiltration can be effectively used to predict the chemotherapy sensitivity of patients. Pfannstiel et al. confirmed that patients presenting with a subtype rich in tumor-infiltrating lymphocytes exhibited an improved response to chemotherapy [17].

The transforming growth factor *β* signaling pathway (TGFB) can demonstrate a suppressive effect on tumors [18]. Studies have shown that the TGFB pathway is capable of inhibiting tumor cell division and proliferation by suppressing c-Myc expression [19]. Zhang et al. found that high expression of TGFB1 in the cancer-associated fibroblasts (CAFs) of SCLC patients was correlated with effective inhibition of tumor growth, enhanced sensitivity to radiotherapy, and antitumor immunity [20]. The effect of the TGFB pathway on the chemotherapy sensitivity of SCLC patients has not yet been investigated from the perspective of the activation level of the TGFB pathway. The single sample Gene Set Enrichment Analysis (ssGSEA) algorithm can be used to determine the pathway activation level in each sample, which has been used in several researches to investigate the prognostic impact of the specific pathway activation on the prognosis of patients. For example, Zhou et al. used the ssGSEA score of DDR and its subpathways, to investigate the effect of the DDR pathway activation status on the prognosis of patients with metastatic urothelial carcinoma [21]. Feng et al. used ssGSEA to discover that a high activation status of the chemokine receptor 3 pathway presents as a potential prognostic predictor for patients treated with immune checkpoint inhibitors [22].

In this study, RNA sequencing was performed on SCLC patients receiving platinum-based chemotherapy from the First Affiliated Hospital of Guangzhou Medical University, the Sun Yat-sen University Cancer Center, and the Zhujiang Hospital of Southern Medical University (Local-SCLC). In addition, we also obtained another validation cohort constructed by George et al. [23]. The effect of the TGFB pathway activation level on the chemotherapy prognosis of SCLC patients was investigated based on the ssGSEA algorithm. The immune cell ratios and immune-related genes in SCLC patients were analyzed. The probable mechanism responsible for the TGFB pathway activation improving chemotherapy prognosis was inferred using Gene Set Enrichment Analysis (GSEA), principal component analysis (PCA), and ssGSEA.

## 2. Methods

### 2.1. Acquisition of Expression Profiles and Relevant Information of Clinical Cohorts

We collected samples of SCLC patients treated with platinum-based chemotherapy from the First Affiliated Hospital of Guangzhou Medical University, the Sun Yat-sen University Cancer Center, and the Zhujiang Hospital of Southern Medical University (Local-SCLC) and performed RNA sequencing. Detailed methods of the sample processing can be found in Lin et al.'s study [24]. Another cohort of SCLC patients receiving platinum-based chemotherapy was obtained from George et al.'s study (George-SCLC) [23]. Samples with both clinical information and RNA-seq data were selected for inclusion in the study. The Local-SCLC cohort and the George-SCLC cohort included 45 and 68 patients enrolled in this study, respectively.

### 2.2. Prognostic Analysis and Drug Sensitivity Prediction

Genes contained in the transforming growth factor *β* signaling pathway (TGFB) were obtained from the MSigDB database [25] (Supplementary Table 1). The ssGSEA analysis was performed using the GSVA R package [26] to determine the activation level of the TGFB pathway based on the obtained ssGSEA score. SCLC patients were divided into a low activation group (TGFB-LOW) and a high activation group (TGFB-HIGH) based on the median of their ssGSEA score. Kaplan-Meier (K-M) survival analysis was used to determine the prognostic impact of the TGFB pathway activation. Based on the sample transcriptome data, the pRRophetic algorithm [27] was used to predict the response to cisplatin treatment for each sample.

### 2.3. Analysis of Immune Infiltration and Metabolic Activity

The IOBR package [28] was used to perform EPIC, MCPcounter, and xCell to analyze the infiltration of immune cells in the immune microenvironment. In addition, the IOBR R package contains many gene sets related to tumor immunity and cellular metabolism. We used the IOBR package to perform the PCA algorithm in order to determine the differences between TGFB-HIGH and TGFB-LOW groups in these gene sets.

### 2.4. Enrichment Analysis

After sequencing the genes according to the logFC values obtained from differential expression analysis using the limma R package [29], GSEA analysis was then performed using the clusterProfiler R package [30]. In addition, the GSVA R package [26] was used to perform ssGSEA analysis. The entries in Gene Ontology (GO), Kyoto Encyclopedia of Genes and Genomes (KEGG), and Reactome were used for analysis. Entries with a value of *p* < 0.05 were considered statistically significant.

### 2.5. Statistical Analysis

**Figure 1:**
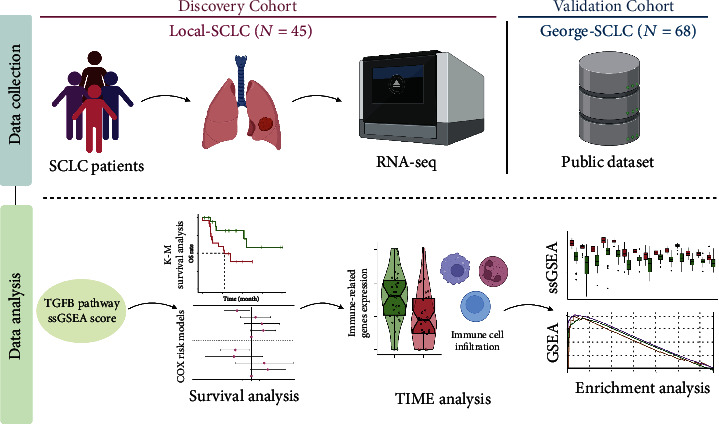
Flow chart of this study.

**Figure 2:**
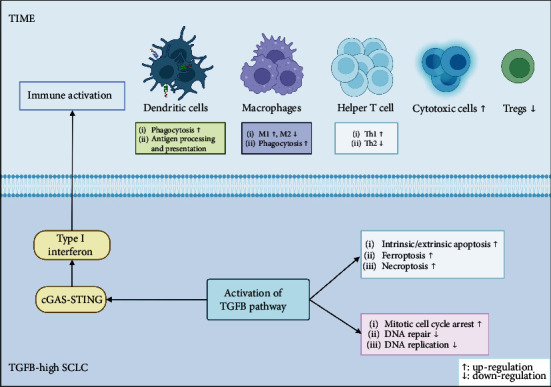
Schematic representation of possible mechanisms by which activation of the TGFB pathway improves prognosis of chemotherapy in SCLC patients.

The Mann–Whitney *U* test was used to compare continuous variables between the two groups. The Kaplan-Meier (K-M) method and log-rank test were used in the survival analysis. Univariate and multivariate COX proportional risk models were used to determine the effect of clinically relevant parameters and TGFB pathway activation scores on prognosis. The time-dependent-ROC was conducted by the timeROC R package [31]. Spearman correlation coefficient is used in correlation analysis. Values whereby *p* < 0.05 were considered statistically significant. Boxplots were drawn using the ggplot2 R package [32]. All statistical tests were two-sided. Figures 1 and 2 were created with BioRender (BioRender.com). Statistical tests and visual analyses were conducted using R software (version 4.1.2).

## 3. Results

### 3.1. Activation of the TGF-*β* Signaling Pathway Is Associated with an Improved Prognosis in SCLC Patients

**Figure 3:**
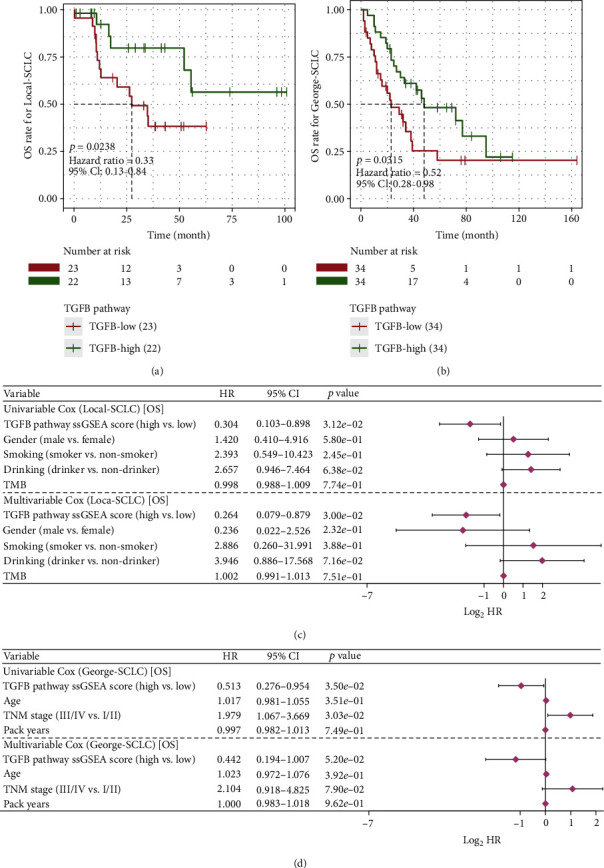
Effect of transforming growth factor *β* signaling pathway activation levels on the prognosis of chemotherapy in SCLC patients. (a) K-M survival analysis in the Local-SCLC cohort. (b) K-M survival analysis in the George-SCLC cohort. (c) Univariate and multivariate COX regression models for the Local-SCLC cohort. (d) Univariate and multivariate COX regression models for the George-SCLC cohort.

The flowchart of our work is presented in Figure 1. SCLC patients in both cohorts were divided into a low activation group (TGFB-LOW) and a high activation group (TGFB-HIGH) according to the ssGSEA score of the TGFB pathway. Clinical statistics between the TGFB-LOW and TGFB-HIGH groups in the Local-SCLC cohort and George-SCLC cohort were found not to be statistically different (*p* > 0.05, Supplementary Tables 2 and 3). Survival analysis showed that in both cohorts, the TGFB-HIGH group exhibited better overall survival (OS) (Local-SCLC: hazard ratio = 0.0238, (95% CI, 0.13-0.84), *p* = 0.0238, Figure 3(a); George-SCLC: hazard ratio = 0.0315, (95% CI, 0.28-0.98), *p* = 0.0315, Figure 3(b)). The TGFB pathway activation status and clinical information were included in univariate and multivariate COX models. In the Local-SCLC cohort, activation of the TGFB pathway was an independent risk factor for prognosis (*p* = 0.03, Figure 3(c)). In the George-SCLC cohort, the univariate COX model showed that high activation of the TGFB pathway was associated with an improved prognosis in SCLC patients treated with platinum-based chemotherapy (*p* = 0.035, Figure 3(d)). Time-dependent-ROC shows that the 1- and 3-year AUCs of TGFB ssGSEA score are 0.755 and 0.785 in Local-SCLC and 0.630 and 0.601 in George-SCLC, respectively (Supplementary Figure 1). We also analyzed the impact of genes in the TGFB pathway on the OS of SCLC patients. In the Local-SCLC cohort, most of the genes in the TGFB had a protective effect on OS; however, in the George-SCLC cohort, only two genes had a statistically significant protective effect on OS (Supplementary Table 4). Furthermore, the TGFB-HIGH group exhibited significantly lower estimated IC50 values to platinum-based chemotherapeutic agents in the George-SCLC cohort (*p* < 0.05, Supplementary Figure 2).

### 3.2. Analysis of Iimmune-Rrelated Ggenes and Iimmune Iinfiltration

**Figure 4:**
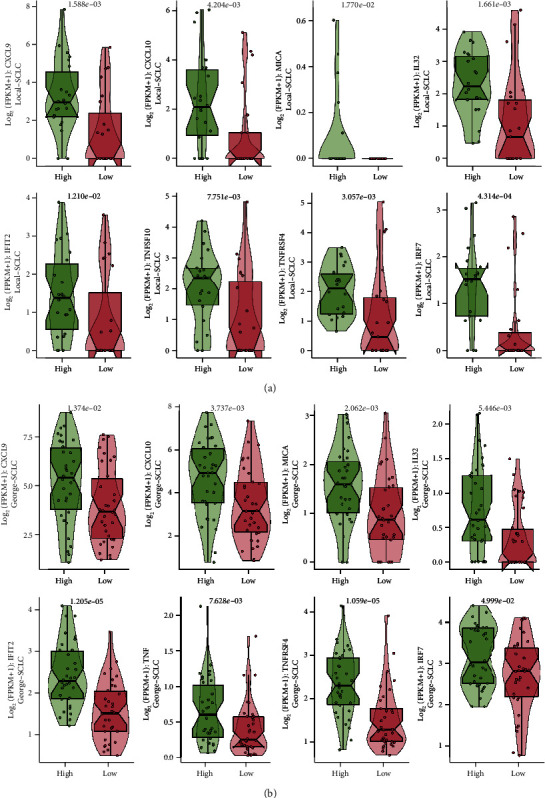
Comparison of the immune-related gene expression in SCLC patients. (a) Expression of immune-related genes in the Local-SCLC cohort. (b) Expression of immune-related genes in the George-SCLC cohort.

We compared the expression of some immune-related genes between the two groups. In the Local-SCLC cohort, we observed a high expression of genes including CXCL9, CXCL10, MICA, IFIT2, TNFRSF4, and IRF7, in the TGFB-HIGH group (all *p* < 0.05, Figure 4(a)). Similar results were observed in the George-SCLC cohort (all *p* < 0.05, Figure 4(b)). We further analyzed the coexpression of genes in the TGFB pathway with immune-related genes and could find that genes within the TGFB pathway had a strong positive correlation with immune-related genes in Local-SCLC cohort (Supplementary Figure 3A, *p* < 0.05), while in the George-SCLC cohort, this strong positive correlation was mainly occurred in RHOA, TGFB1, and TGFBR2 (Supplementary Figure 3B, *p* < 0.05).

**Figure 5:**
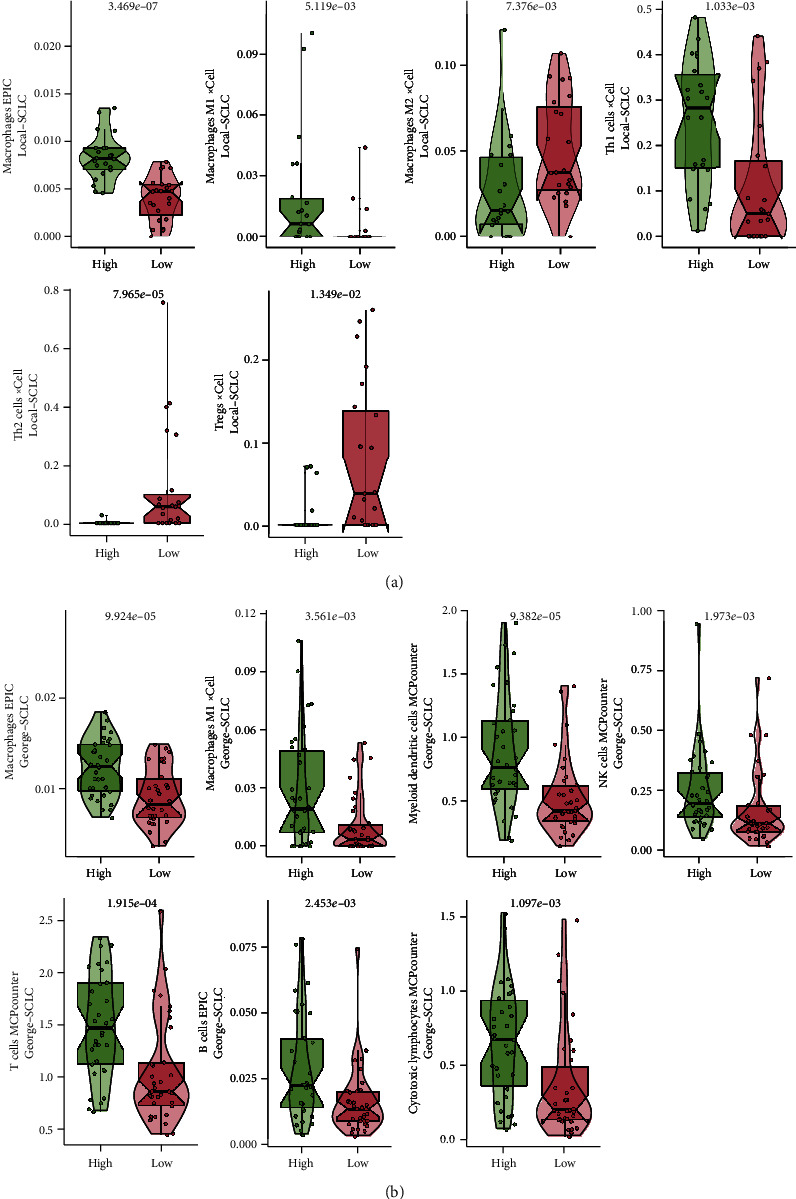
Comparison of immune cell infiltration in SCLC patients. (a) Immune cell infiltration in the Local-SCLC cohort. (b) Immune cell infiltration in the George-SCLC cohort.

**Figure 6:**
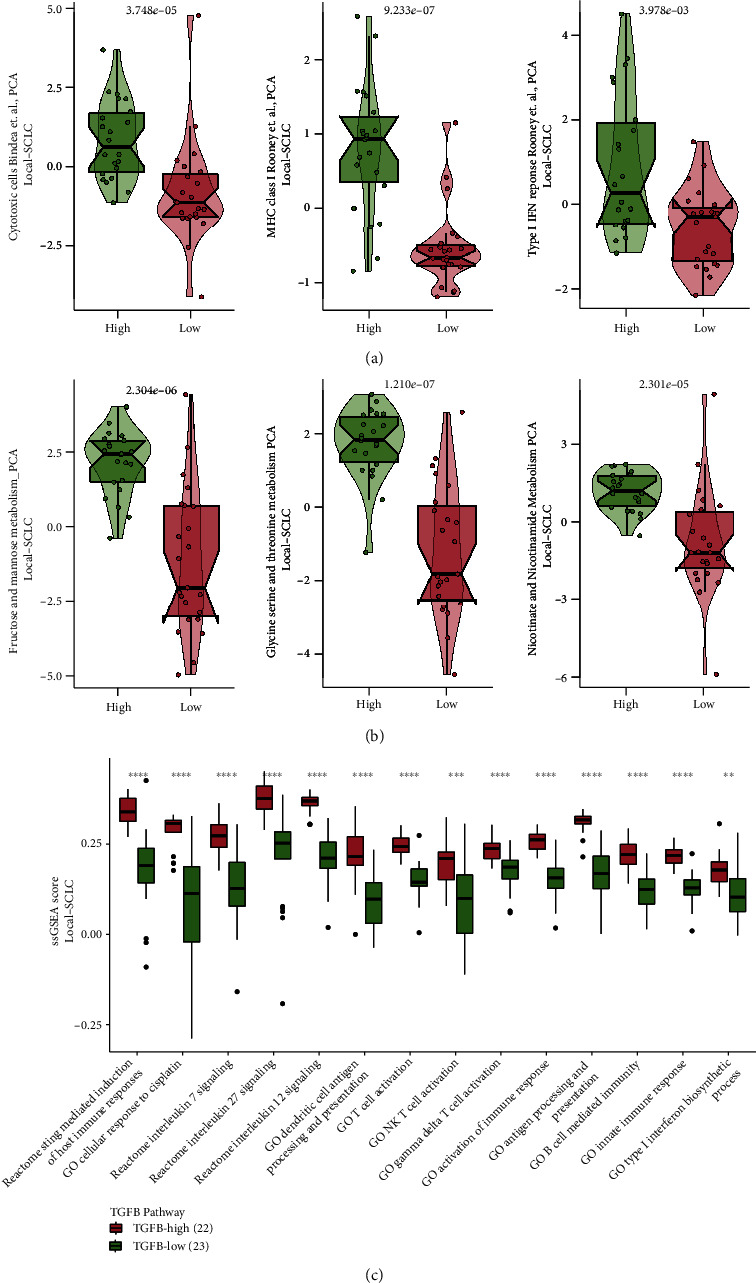
PCA and ssGSEA analyses in SCLC patients in the Local-SCLC cohort. (a) PCA analysis of immune-related pathways in the Local-SCLC cohort. (b) PCA analysis of metabolism-related pathways in the Local-SCLC cohort. (c) Results of ssGSEA in the Local-SCLC cohort. ^∗^*p* < 0.05; ^∗∗^*p* < 0.01; ^∗∗∗^*p* < 0.001; ^∗∗∗∗^*p* < 0.0001.

Next, we evaluated immune cell infiltration in the two groups. In the Local-SCLC cohort, macrophages, M1 macrophages, and Th1 cells were found to be higher in the TGFB-HIGH group than in the TGFB-LOW group, while Th2 cells, Tregs, and M2 macrophages were lower than in the TGFB-LOW group (all *p* < 0.05, Figure 5(a)). In the George-SCLC cohort, there were more B cells, CD4+ T cells, M1 macrophages, NK cells, and myeloid dendritic cells in the TGFB-HIGH group than in the TGFB-LOW group (all *p* < 0.05, Figure 5(b)). PCA analysis based on the IOBR package built-in immune-related gene set revealed that the TGFB-HIGH groups in both cohorts had higher cytotoxic cells, MHC class I, and type I IFN response than the TGFB-LOW groups (all *p* < 0.05, Figure 6(a); Supplementary Figure 4A).

### 3.3. Pathway Enrichment Analysis and Metabolic Activity Analysis

Functional enrichment analysis was performed on both cohorts. The ssGSEA results showed that the cellular response to cisplatin, STING-mediated induction of host immune response, activation of immune response, type I interferon biosynthetic process, antigen processing and presentation, and multiple immune cell activation pathways were all significantly upregulated in the TGFB-HIGH group (all *p* < 0.05, Figure 6(c), Supplementary Figure 4C).

**Figure 7:**
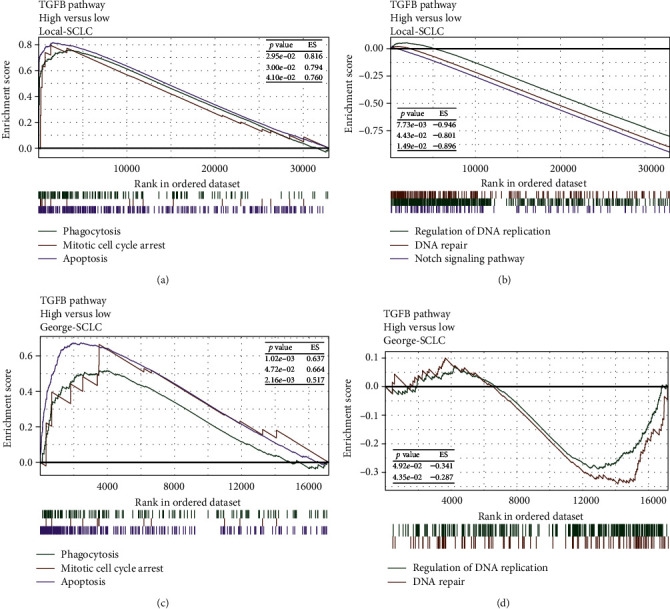
Enrichment analysis results for SCLC patients. (a, b) Results of GSEA in the Local-SCLC cohort. (c, d) Results of GSEA in the George-SCLC cohort. (c) Results of ssGSEA in the Local-SCLC cohort. (d) Results of ssGSEA in the George-SCLC cohort.

The GSEA results indicated that in the Local-SCLC cohort, pathways such as mitotic cell cycle arrest, apoptosis, phagocytosis, ferroptosis, necroptosis, and intrinsic and extrinsic apoptosis were all significantly upregulated in the TGFB-HIGH group (*p* < 0.05, *ES* > 0, Figure 7(a), Supplementary Figure 5A-B), while DNA repair, DNA replication regulation, and the notch signaling pathway were significantly downregulated in the TGFB-HIGH group (*p* < 0.05, *ES* < 0, Figure 7(b)). The GSEA enrichment results in the George-SCLC cohort validated our findings in the Local-SCLC cohort (*p* < 0.05, *ES* > 0, Figure 7(c); *p* < 0.05, *ES* < 0, Figure 7(d)). The role of tumor metabolism in chemotherapy resistance has attracted increasing attention in recent years [33, 34]. We examined the differences in the metabolism-related gene sets built into the IOBR package in two cohorts. We observed that glycine serine and threonine metabolism, fructose and mannose metabolism, and nicotinate and nicotinamide metabolism were upregulated in the TGFB-HIGH group in both cohorts (all *p* < 0.05, Figure 6(b), Supplementary Figure 4B).

## 4. Discussion

Our study revealed that a high activation status of the TGFB pathway was significantly associated with a better prognosis for SCLC patients treated with platinum-based chemotherapy. We analyzed the expression of immune-related genes, as well as immune cell infiltration within SCLC patients. We found that patients in the TGFB-HIGH group were characterized by an antitumor immune microenvironment that facilitated and enhanced the cytotoxicity of platinum-based chemotherapeutic agents against cancer. Enrichment analysis further indicated that the improved prognosis observed in the TGFB-HIGH group might be attributed to the STING-mediated type I interferon secretion and resultant immune activation, in conjunction with a weakened capacity for DNA repair and enhanced apoptosis, all of which subsequently promoted the sensitivity of SCLC to platinum-based chemotherapy. Therefore, we infer that a high activation status of the TGFB pathway may enhance the sensitivity of SCLC patients to platinum-based chemotherapy by activating immunity, inhibiting repair, and promoting apoptosis (Figure 2).

A high activation status of the TGFB pathway is characterized by an immune-related gene expression pattern that favors chemotherapy. We observed high expression of specific immune-related genes (e.g., CXCL9, CXCL10, and TNF) in the TGFB-HIGH group. Previous studies have pointed out the relationship between these genes and the efficacy of platinum-based chemotherapy. CXCL9 [35] and CXCL10 [36] can effectively inhibit angiogenesis, enhance apoptosis and immune infiltration of tumor cells, and enhance antitumor immunity in platinum-based chemotherapy drug treatment. Wu et al. [37] demonstrated in vitro that MICA could augment the antitumor activity of chemotherapeutic drugs by enhancing the tumor-killing activity of both NK and T cells. In addition, studies have shown that IL-32 [38] and TNF [39] could enhance cisplatin-induced apoptosis. Upregulation of IFIT2 reversed the cisplatin-resistant phenotype [40]. Moreover, patients with high TNFSF10 expression usually exhibited a good response to chemotherapy [41], while TNFRSF4 was associated with chemosensitivity and good prognosis in ovarian cancer patients [42], and IRF7 was upregulated in patients in the chemotherapy responsive group [43]. IL-7, IL-12, and IL-27 signaling pathways were highly activated in the TGFB-HIGH group, and studies have shown that they can enhance the antitumor response of chemotherapeutic drugs [44–46]. Thus, a high activation status of the TGFB pathway may improve the response to chemotherapy in SCLC patients by affecting the expression of immune-related genes.

A high activation of the TGFB pathway expresses an immune microenvironment conducive to the antitumor effect observed in chemotherapy drugs. The TIME can influence the antitumor effects of chemotherapeutic drugs [13, 14]. The results of our study showed that SCLC patients in the TGFB-HIGH group were characterized by immune activation and differential immune cell infiltration. *γ*/*δ* T cells can enhance carboplatin-induced cytotoxicity against advanced bladder cancer cells [47]. Cytotoxic lymphocytes and NK cells can inhibit tumor progression by secreting perforin and granzyme to kill abnormal cells [48, 49]. Tregs can suppress the antitumor immune response, and a low degree of Treg infiltration in the TGFB-HIGH group facilitates the elimination of its immunosuppressive effect [14, 50]. With regards to helper T cells, the upregulation of Th1 and downregulation of Th2 in the TGFB-HIGH group assist chemotherapeutic drugs in fighting against tumor cells [50]. Different phenotypes of macrophages exert different effects on chemotherapeutic outcomes. We observed more M1 macrophages and fewer M2 macrophages in the TGFB-HIGH group compared with the TGFB-LOW group in the Local-SCLC. In general, M1 macrophages inhibit tumor growth, while M2 macrophages promote tumor progression by suppressing the immune response [51, 52]. Finally, B cell infiltration was positively correlated with prolonged disease-free survival in patients receiving chemotherapy [53]. Based on the above research results, we speculated that the antitumor immune microenvironment characteristics observed in the TGFB-HIGH group might contribute to the efficacy and activity of platinum-based chemotherapy to eradicating tumors.

The inhibition of DNA repair, in conjunction with the enhanced apoptosis status observed in the TGFB-HIGH group, augments the antitumor effects of platinum-based chemotherapeutic agents. The results of the GSEA enrichment analysis suggest that the TGFB-HIGH group is characterized by cell cycle arrest, decreased ability to repair DNA damage, and enhanced multiple cell death pathways and greater phagocytosis. In terms of the affiliated therapeutic mechanism, cisplatin damages tumor cell DNA through the formation of platinum-DNA adducts, leading to cell cycle arrest and replication inhibition. If DNA damage repair-related pathways can successfully repair DNA damage caused by platinum-based chemotherapy, tumors can avoid death and thus develop chemoresistance. Conversely, weak DNA damage repair capacity results in cells being unable to complete DNA damage repair, which in turn leads to the death of tumor cells in various forms, resulting in tumor sensitivity to chemotherapy drugs and thus exerting an antitumor effect [11, 54]. Therefore, in this study, the low DNA repair function and increased apoptosis observed in the TGFB-HIGH group facilitated the antitumor effects of cisplatin in patients with SCLC.

The high activation status of the TGFB pathway may enhance the antitumor effects of platinum-based chemotherapy through activating the STING pathway. The ssGSEA analysis suggested that SCLC patients in the TGFB-HIGH group presented with both an upregulated response to cisplatin as well as a high activation status in the STING-mediated immune response pathway. Studies have shown that platinum-based chemotherapy can enhance the antitumor immune response by activating the STING pathway [55]. The cGAS senses the presence of abnormal DNA in the cytoplasm and can then convert to cGAMP to allow it to bind to STING. The cGAMP-STING complex then activates the TBK1-IRF3 signaling axis to induce the secretion of type I interferon, which in turn activates innate immunity. Type I interferon incorporates multiple anticancer properties and can therefore successfully inhibit tumor progression [56]. As antigen-presenting cells, DCs can present antigens to T cells and drive the formation of tumor antigen-specific T cells. STING activation induces the production of type I interferons that enhance their antitumor efficacy by enriching the costimulatory activity of DCs and enhancing their ability to cross-present antigens to T cells [57]. Therefore, we inferred that the improved prognosis in the TGFB-HIGH group was closely related to STING-mediated immune activation.

Metabolic changes in the TGFB-HIGH group may also be a possible cause of chemosensitivity in SCLC patients. Zhao et al. found that inhibition of serine metabolism could reduce the toxic and proapoptotic effects of platinum-based chemotherapy on gastric cancer cells through increasing chromatin density, thereby promoting drug resistance [58]. Gonzalez et al. demonstrated that the combination of mannose with conventional chemotherapy can render tumor cells sensitive to chemotherapy by affecting levels of antiapoptotic proteins [59]. Wei et al. found that nicotinamide reversed the resistance of breast cancer cells to Adriamycin by inhibiting the SIRT1/Akt pathway [60]. Therefore, metabolic changes may also serve to explain the resultant improved prognosis of chemotherapy treatment in SCLC patients with regards to the high activation status of the TGFB pathway.

## 5. Conclusion

In this study, we found that a high activation status of the TGFB pathway could be used as a biomarker to predict sensitivity to platinum-based chemotherapy in SCLC patients. The TGFB-HIGH group exhibited a different immune-related gene pattern and immune cell infiltration status to the TGFB-LOW group. Mechanistic analysis showed that the improved prognosis of SCLC patients in the TGBF-HIGH group was associated with increased STING-mediated type I IFN secretion, enhanced antitumor immunity, reduced DNA damage repair, and enhanced apoptosis.

## Data Availability

All the data generated or analyzed during this study are included in this published article (https://www.cbioportal.org/study/summary?id=sclc_ucologne_2015). All other relevant data are available from the authors of this study upon request.

## Abbreviations

TGFB: Transforming growth factor *β* signaling pathway
TGFB-HIGH: High activation status of transforming growth factor *β* signaling pathway
TGFB-LOW: Low activation status of transforming growth factor *β* signaling pathway
SCLC: Small cell lung cancer
TIME: Tumor immune microenvironment
GSEA: Gene Set Enrichment Analysis
ssGSEA: Single sample Gene Set Enrichment Analysis
PCA: Principal component analysis
KEGG: Kyoto Encyclopedia of Genes and Genomes
GO: Gene Ontology
OS: Overall survival
NK: Natural killer
Tregs: Regulatory T cells
IFN: Interferon
DCs: Dendritic cells
CAFs: Cancer-associated fibroblasts
Th: Helper T cell
IL: Interleukin
ROC: Receiver operating characteristic
AUC: Area under curve.

## Supplementary Materials

Supplementary 1 Supplementary Table 1: gene names contained in the transforming growth factor *β* signaling pathway.

Supplementary 2 Supplementary Table 2: clinical demographics of SCLC patients in the Local-SCLC cohort in both the TGFB-HIGH and TGFB-LOW groups.

Supplementary 3 Supplementary Table 3: clinical demographics of SCLC patients in the George-SCLC cohort in both the TGFB-HIGH and TGFB-LOW groups.

Supplementary 4 Supplementary Table 4: univariate COX regression analysis of genes within the TGFB pathway in both cohorts.

Supplementary 5 Supplementary Figure 1: time-dependent ROC analysis for predicting 1- and 3-year OS in patients with SCLC in Local-SCLC cohort (a) and George-SCLC cohort (b).

Supplementary 6 Supplementary Figure 2: predicted IC50 values for SCLC patients in the George-SCLC cohort.

Supplementary 7 Supplementary Figure 3: correlation between genes in TGFB pathway and immune-related genes in Local-SCLC cohort (a) and George-SCLC cohort (b). ^∗^*p* < 0.05; ^∗∗^*p* < 0.01; ^∗∗∗^*p* < 0.001; ^∗∗∗∗^*p* < 0.0001.

Supplementary 8 Supplementary Figure 4: PCA and ssGSEA analyses in SCLC patients in the George-SCLC cohort. (a) PCA analysis of immune-related pathways in the George-SCLC cohort. (b) PCA analysis of metabolism-related pathways in the George-SCLC cohort. (c) Results of ssGSEA in the George-SCLC cohort. ^∗^*p* < 0.05; ^∗∗^*p* < 0.01; ^∗∗∗^*p* < 0.001; ^∗∗∗∗^*p* < 0.0001.

Supplementary 9 Supplementary Figure 5: GSEA enrichment results in the Local-SCLC cohort not shown in the main text.
