# Supplementary material for: Activation of the TGF-β Pathway Enhances the Efficacy of Platinum-Based Chemotherapy in Small Cell Lung Cancer Patients
Source: Dis Markers. 2022 Dec 21;2022:8766448. doi: 10.1155/2022/8766448 (PMC9798106; doi:10.1155/2022/8766448)
Supplement: Supplementary 1 — Supplementary Table 1: gene names contained in the transforming growth factor β signaling pathway. [file 8766448.f1.pdf]

Supplementary TABLE 1. List of genes contained in the transforming growth factor β signaling pathway for analysis.

TGFB1
TGFBR1
TGFBR2
ARHGEF18
CGN
F11R
FKBP1A
PARD3
PARD6A
PRKCZ
RHOA
RPS27A
SMURF1
UBA52
UBB
UBC
