# Supplementary material for: Activation of the TGF-β Pathway Enhances the Efficacy of Platinum-Based Chemotherapy in Small Cell Lung Cancer Patients
Source: Dis Markers. 2022 Dec 21;2022:8766448. doi: 10.1155/2022/8766448 (PMC9798106; doi:10.1155/2022/8766448)
Supplement: Supplementary 2 — Supplementary Table 2: clinical demographics of SCLC patients in the Local-SCLC cohort in both the TGFB-HIGH and TGFB-LOW groups. [file 8766448.f2.pdf]

Supplementary TABLE 2 Clinical demographics of SCLC patients in the Local-SCLC cohort in the TGFB-HIGH and TGFB-LOW groups.

	**TGFB-HIGH(N=22)**	**TGFB-LOW(N=23)**	**Overall (N=45)**	**P value**
**Gender**				0.4591
Female	5(22.7%)	3(13.0%)	8(17.8%)	
Male	17(77.3%)	20(87.0%)	37(82.2%)	
**Smoking**				0.0689
Non-Smoker	8(36.4%)	2(8.7%)	10(22.2%)	
Smoker	14(63.6%)	20(87.0%)	34(75.6%)	
Missing	0(0%)	1(4.3%)	1(2.2%)	
**Drinking**				0.7626
Drinker	11(50.0%)	13(56.5%)	24(53.3%)	
Non-Drinker	11(50.0%)	9(39.1%)	20(44.4%)	
Missing	0(0%)	1(4.3%)	1(2.2%)	
**TMB**				0.7106
Mean (SD)	57.4(55.9)	40.4(33.3)	48.7(46.0)	
Median [Min,Max]	30.9[2.82,192]	30.4[9.92,165]	30.4[2.82,192]	
