# Supplementary material for: Activation of the TGF-β Pathway Enhances the Efficacy of Platinum-Based Chemotherapy in Small Cell Lung Cancer Patients
Source: Dis Markers. 2022 Dec 21;2022:8766448. doi: 10.1155/2022/8766448 (PMC9798106; doi:10.1155/2022/8766448)
Supplement: Supplementary 3 — Supplementary Table 3: clinical demographics of SCLC patients in the George-SCLC cohort in both the TGFB-HIGH and TGFB-LOW groups. [file 8766448.f3.pdf]

Supplementary TABLE 3 Clinical demographics of SCLC patients in the George-SCLC cohort in the TGFB-HIGH and TGFB-LOW groups.

	**TGFB-HIGH(N=34)**	**TGFB-LOW(N=34)**	**Overall(N=68)**	**P value**
**TNM stage**				0.3454
I	12(35.3%)	15(44.1%)	27(39.7%)	
II	10(29.4%)	4(11.8%)	14(20.6%)	
III	9(26.5%)	10(29.4%)	19(27.9%)	
IV	3(8.8%)	5(14.7%)	8(11.8%)	
**Age**				0.4500
Mean (SD)	64.9(8.54)	64.1(9.31)	64.5(8.87)	
Median [Min, Max]	65.5[47.0,83.0]	62.5[47.0,83.0]	64.0[47.0,83.0]	
**Pack-Years**				0.6462
Mean (SD)	45.7(27.5)	43.1(23.9)	44.4(25.5)	
Median [Min,Max]	50.0[0,100]	40.0[0.750,90.0]	45.0[0,100]	
Missing	10(29.4%)	11(32.4%)	21(30.9%)	
