# Supplementary material for: Activation of the TGF-β Pathway Enhances the Efficacy of Platinum-Based Chemotherapy in Small Cell Lung Cancer Patients
Source: Dis Markers. 2022 Dec 21;2022:8766448. doi: 10.1155/2022/8766448 (PMC9798106; doi:10.1155/2022/8766448)
Supplement: Supplementary 4 — Supplementary Table 4: univariate COX regression analysis of genes within the TGFB pathway in both cohorts. [file 8766448.f4.pdf]

Supplementary TABLE 4: Univariate COX regression analysis of genes within the TGFB pathway in both in both cohorts.

Gene	Local-SCLC			George-SCLC		
	HR	95%Cl	pvalue	HR	95%Cl	pvalue
**TGFBR2**	0.080023	0.02-0.36	0.000992	1.090708	0.59-2.02	0.783032
**TGFB1**	0.11157	0.03-0.49	0.003669	0.725363	0.39-1.34	0.307455
**SMURF1**	0.151632	0.05-0.48	0.001293	0.649213	0.35-1.21	0.175586
**PRKCZ**	0.197115	0.06-0.61	0.004775	0.629353	0.34-1.17	0.142656
**RPS27A**	0.22354	0.08-0.66	0.006313	0.853177	0.46-1.59	0.618443
**UBA52**	0.295861	0.11-0.82	0.018674	1.14953	0.62-2.13	0.657644
**TGFBR1**	0.300096	0.11-0.85	0.023744	0.83349	0.45-1.54	0.562009
**RHOA**	0.351258	0.13-0.97	0.044179	1.177611	0.64-2.18	0.603197
**FKBP1A**	0.359114	0.13-1	0.049219	2.300245	1.19-4.44	0.013137
**F11R**	0.384509	0.14-1.04	0.058533	1.118657	0.61-2.07	0.720606
**UBC**	0.418293	0.15-1.17	0.096284	1.042809	0.56-1.94	0.894791
**PARD6A**	0.443732	0.17-1.17	0.100128	0.985119	0.53-1.83	0.962012
**ARHGEF18**	0.446151	0.17-1.19	0.107546	0.301425	0.15-0.6	0.000557
**CGN**	0.545439	0.21-1.41	0.210962	0.774417	0.42-1.43	0.41569
**UBB**	0.643221	0.25-1.66	0.363088	1.013048	0.55-1.8	0.967038
**PARD3**	1.074953	0.43-2.71	0.878393	0.60044	0.26-1.36	0.222132
