# Supplementary figures and images for: Activation of the TGF-β Pathway Enhances the Efficacy of Platinum-Based Chemotherapy in Small Cell Lung Cancer Patients
Source: Dis Markers. 2022 Dec 21;2022:8766448. doi: 10.1155/2022/8766448 (PMC9798106; doi:10.1155/2022/8766448)

**A**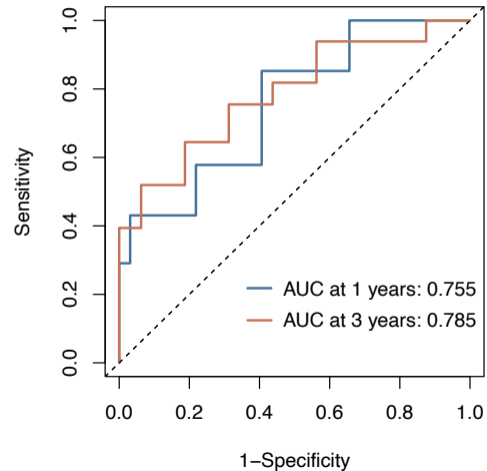**B**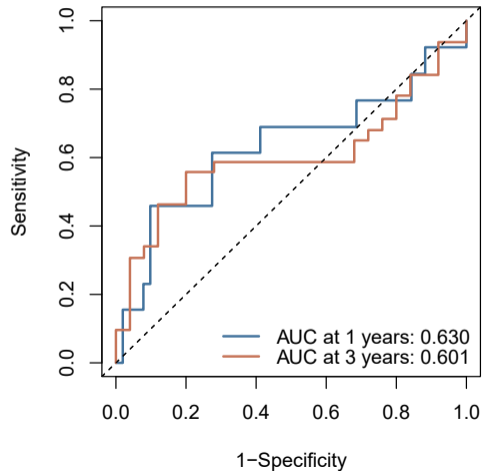

Supplement: Supplementary 5 — Supplementary Figure 1: time-dependent ROC analysis for predicting 1- and 3-year OS in patients with SCLC in Local-SCLC cohort (a) and George-SCLC cohort (b). [file 8766448.f5.pdf]

Estimated IC50 of Cisplatin (Geroge-SCLC)

$2.882 \times 10^{-2}$

7  
6  
5  
4  
3  
2

High

Low

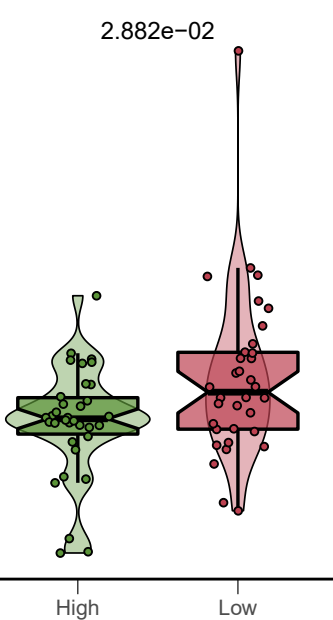

Supplement: Supplementary 6 — Supplementary Figure 2: predicted IC50 values for SCLC patients in the George-SCLC cohort. [file 8766448.f6.pdf]

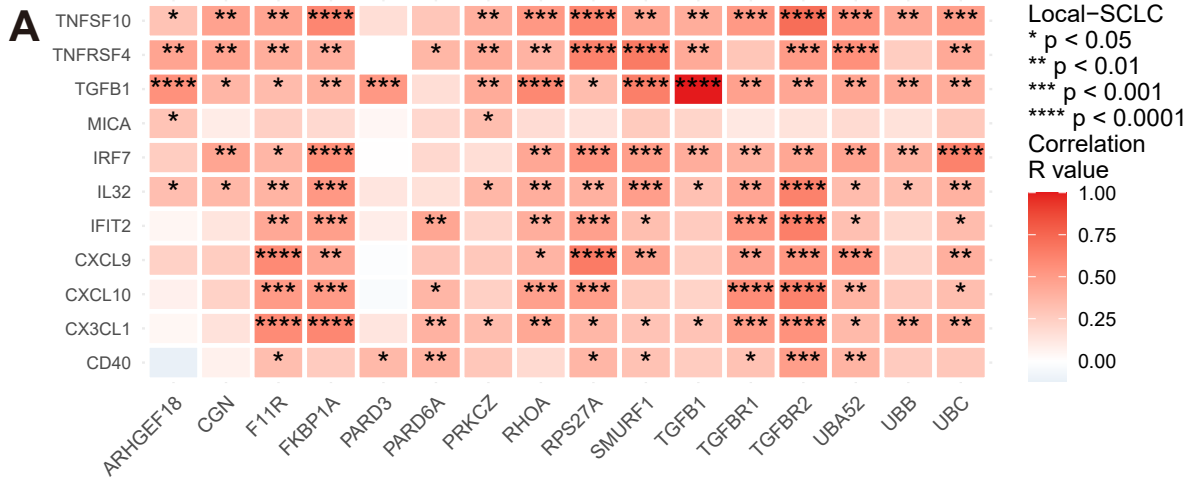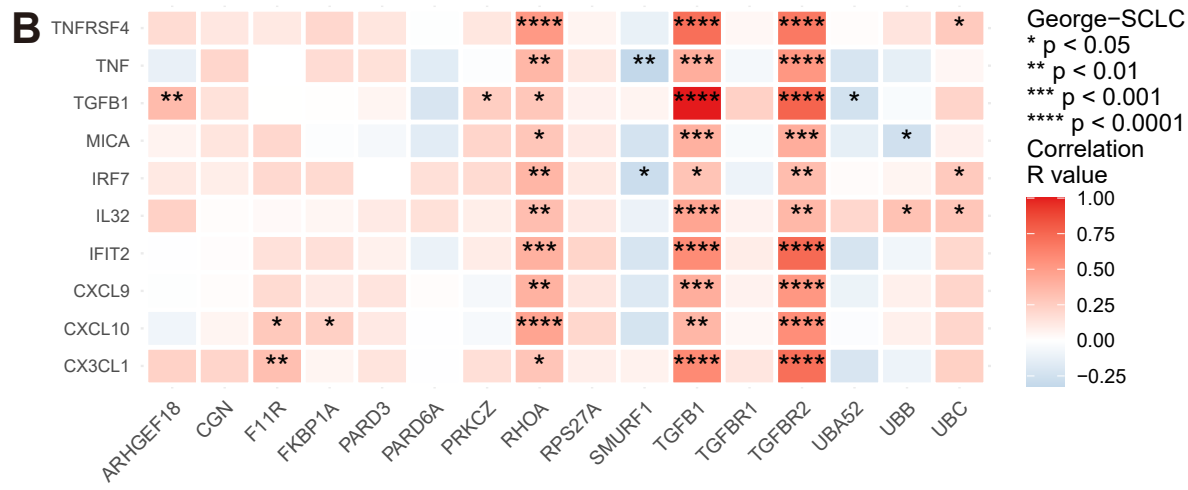

Supplement: Supplementary 7 — Supplementary Figure 3: correlation between genes in TGFB pathway and immune-related genes in Local-SCLC cohort (a) and George-SCLC cohort (b). ∗p < 0.05; ∗∗p < 0.01; ∗∗∗p < 0.001; ∗∗∗∗p < 0.0001. [file 8766448.f7.pdf]

**A**

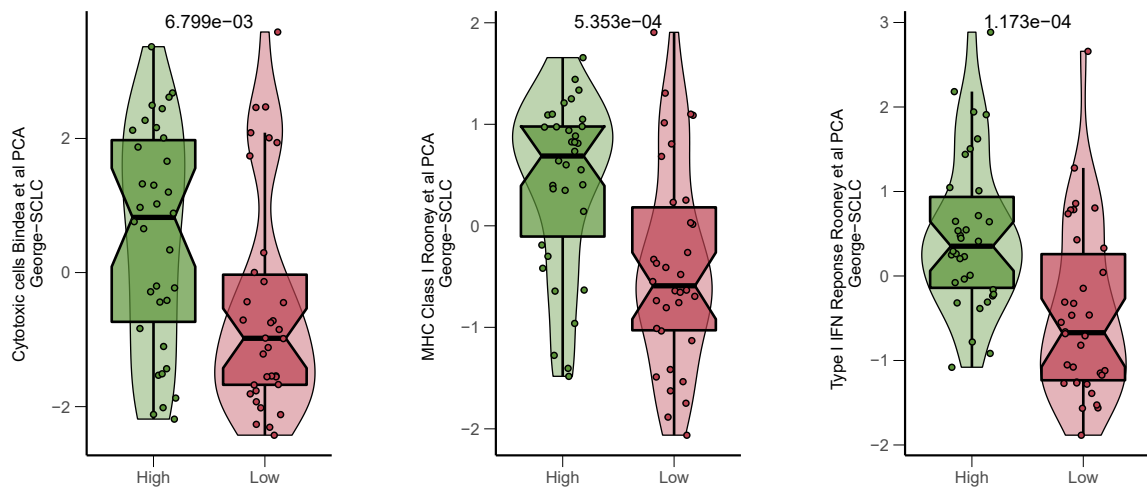

**B**

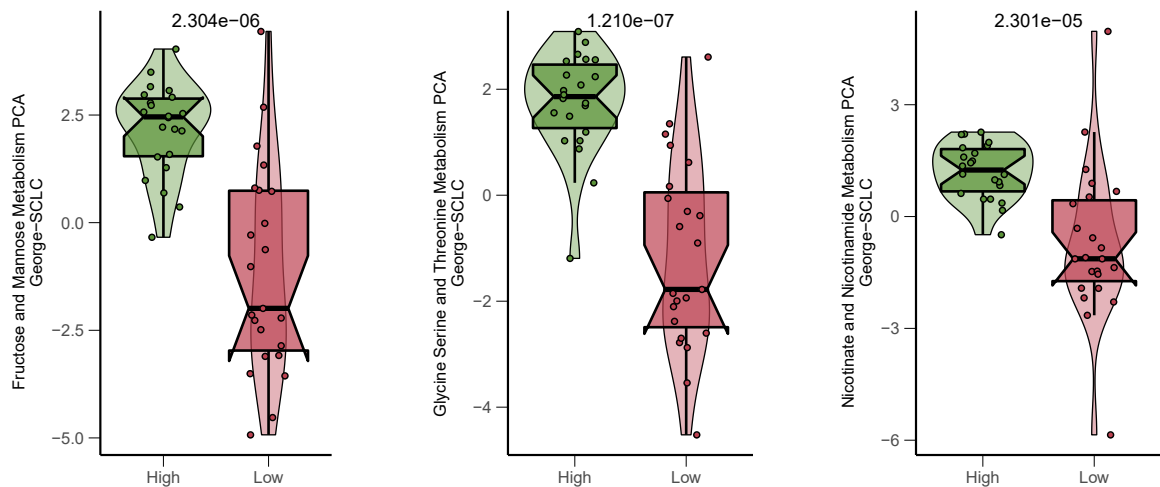

**C**

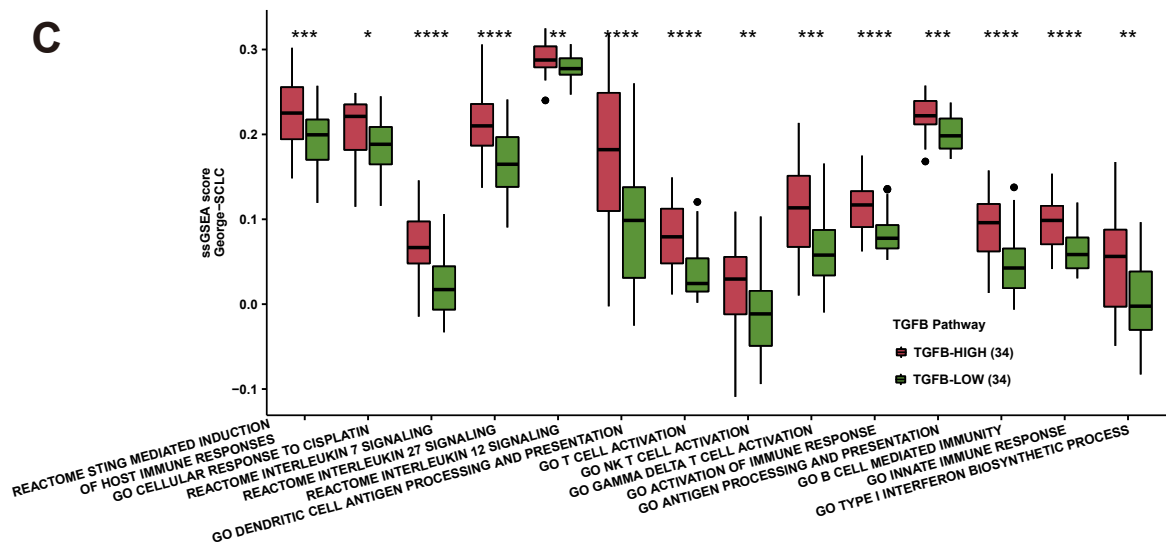

Supplement: Supplementary 8 — Supplementary Figure 4: PCA and ssGSEA analyses in SCLC patients in the George-SCLC cohort. (a) PCA analysis of immune-related pathways in the George-SCLC cohort. (b) PCA analysis of metabolism-related pathways in the George-SCLC cohort. (c) Results of ssGSEA in the George-SCLC cohort. ∗p < 0.05; ∗∗p < 0.01; ∗∗∗p < 0.001; ∗∗∗∗p < 0.0001. [file 8766448.f8.pdf]

**A**

TGFB Pathway  
High versus Low  
Local-SCLC

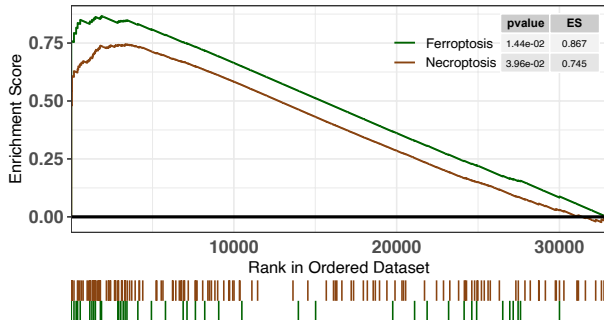**B**

TGFB Pathway  
High versus Low  
Local-SCLC

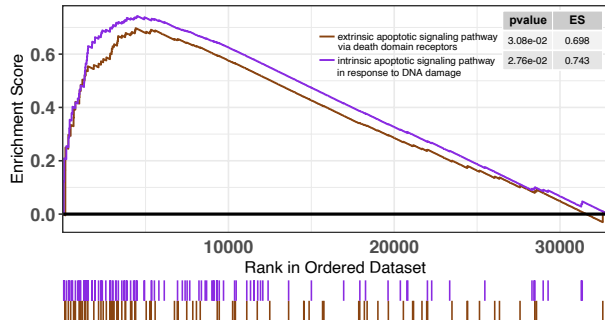

Supplement: Supplementary 9 — Supplementary Figure 5: GSEA enrichment results in the Local-SCLC cohort not shown in the main text. [file 8766448.f9.pdf]
